# Dissecting the Roles of Phosphorus Use Efficiency, Organic Acid Anions, and Aluminum-Responsive Genes under Aluminum Toxicity and Phosphorus Deficiency in Ryegrass Plants

**DOI:** 10.3390/plants13070929

**Published:** 2024-03-23

**Authors:** Leyla Parra-Almuna, Sofía Pontigo, Antonieta Ruiz, Felipe González, Nuria Ferrol, María de la Luz Mora, Paula Cartes

**Affiliations:** 1 Center of Plant Soil Interaction and Natural Resources Biotechnology, Scientific and Technological Bioresource Nucleus (BIOREN-UFRO), Universidad de La Frontera, P.O. Box 54-D, Temuco 4811230, Chile; leyla.parra@ufrontera.cl (L.P.-A.); sofia.pontigo@ufrontera.cl (S.P.); 2 Departamento de Ciencias Químicas y Recursos Naturales, Facultad de Ingeniería y Ciencias, Universidad de La Frontera, P.O. Box 54-D, Temuco 4811230, Chile; maria.ruiz@ufrontera.cl; 3 Programa de Doctorado en Ciencias Mención Biología Celular y Molecular Aplicada, Universidad de La Frontera, P.O. Box 54-D, Temuco 4811230, Chile; f.gonzalez31@ufromail.cl; 4 Departamento de Microbiología del Suelo y Sistemas Simbióticos, Estación Experimental del Zaidín, Consejo Superior de Investigaciones Científicas (CSIC), Profesor Albareda 1, 18008 Granada, Spain; nuria.ferrol@eez.csic.es

**Keywords:** aluminum toxicity, phosphorus deficiency, phosphorus use efficiency, aluminum resistance, aluminum-responsive genes, ryegrass

## Abstract

Aluminum (Al) toxicity and phosphorus (P) deficiency are widely recognized as major constraints to agricultural productivity in acidic soils. Under this scenario, the development of ryegrass plants with enhanced P use efficiency and Al resistance is a promising approach by which to maintain pasture production. In this study, we assessed the contribution of growth traits, P efficiency, organic acid anion (OA) exudation, and the expression of Al-responsive genes in improving tolerance to concurrent low-P and Al stress in ryegrass (*Lolium perenne* L.). Ryegrass plants were hydroponically grown under optimal (0.1 mM) or low-P (0.01 mM) conditions for 21 days, and further supplied with Al (0 and 0.2 mM) for 3 h, 24 h and 7 days. Accordingly, higher Al accumulation in the roots and lower Al translocation to the shoots were found in ryegrass exposed to both stresses. Aluminum toxicity and P limitation did not change the OA exudation pattern exhibited by roots. However, an improvement in the root growth traits and P accumulation was found, suggesting an enhancement in Al tolerance and P efficiency under combined Al and low-P stress. Al-responsive genes were highly upregulated by Al stress and P limitation, and also closely related to P utilization efficiency. Overall, our results provide evidence of the specific strategies used by ryegrass to co-adapt to multiple stresses in acid soils.

## 1. Introduction

Acid soils (pH ≤ 5.5) account for more than 50% of the potential arable land worldwide [1,2]. Mineral nutrient imbalance frequently occurs in these soils due to a decrease in the availability of essential elements for plants such as phosphorus (P), while other metal elements, such as aluminum (Al), iron (Fe), and manganese (Mn), become toxic [3].

The availability of inorganic P (Pi; H_2_PO_4_^−^ and HPO_4_^2−^) in acid soils—the primary form acquired by plant roots—is significantly restricted as a consequence of its rapid sorption by mineral surfaces and organic matter, as well as its strong binding with metal cations [4,5]. In addition, under low-pH conditions, high amounts of the Al trivalent ionic form (Al^3+^) become available to plants, negatively affecting a wide range of physical, cellular, and molecular processes [6,7]. Therefore, P deficiency and Al toxicity in acid soils constitute major mineral stressors that limit the growth and productivity of crops [3,8].

To cope with P limitation and Al stress conditions, plants have evolved numerous morphological, physiological and molecular adaptive mechanisms [2]. Accordingly, to enhance the uptake, remobilization, and internal recycling of P under low-P conditions, root system architecture (RSA) modifications, a higher production of root exudates to liberate or solubilize P (e.g., organic acid anions (OAs), protons, and phosphatases), and the improved function of phosphate transporters (PHTs) have been reported [9,10]. All of these strategies enable plants to increase their ability to take up P from the soil (i.e., P acquisition efficiency [PAE]) and use the acquired P to generate biomass or yield (i.e., P utilization efficiency [PUE]) [11,12]. In addition, to cope with Al toxicity, plants use Al external exclusion and Al internal tolerance mechanisms. The first strategy enables plants to release organic molecules (e.g., OAs and phenolic compounds) to form stable and non-phytotoxic chelates with Al in the rhizosphere, while the internal tolerance mechanism allows the detoxification of Al through intracellular Al complexation and subsequent sequestration into vacuoles [13,14].

The interactive effect of P deficiency and Al toxicity in plants has mainly been associated with the induction of OA exudation [15,16]. Nevertheless, other lesser explored mechanisms have been associated with changes in RSA [17,18], the activation of the same transcription factors (TFs) [19,20,21], and phytohormone signalling crosstalk [20].

In recent years, internal Al detoxification based on Al transport has been documented as a key strategy related to Al tolerance in plants [3,15,21,22]. In this context, the initial steps of internal Al detoxification in rice have been related to the function of a unique Al transporter localized at the plasma membrane that belongs to the natural resistance-associated macrophage protein (NRAMP) family, named OsNRAT1 (Al transporter 1) [23]. Interestingly, this protein exclusively transports Al^3+^ from the root cell wall into the root cytosol [24]. Then, after the uptake of Al^3+^ in the cytoplasm, the vacuolar sequestration of Al is facilitated by a tonoplast-localized half-size ABC transporter, i.e., OsALS1 (Al sensitive 1) [25]. The functional and regulatory components underlying these internal Al detoxification processes mediated by Al transporters are mostly studied in model plants such as rice and Arabidopsis; however, in other plant species, this internal strategy remains poorly explored [3,22].

Currently, the evidence has suggested that Al resistance can have pleiotropic effects on P efficiency, highlighting the role of Al resistance in crop adaptation to acid soils [26,27,28,29]. Although this co-adapted mechanism could allow plants to tolerate Al^3+^ and utilize P more efficiently, little is known about the influence of genes related to Al resistance on the improvement of PAE and PUE in plants [26,29].

Perennial ryegrass (*Lolium perenne* L.) is a grass species that is widely cultivated in temperate regions of the world for turf and forage purposes [30]. In southern Chile, it is the dominant forage species supporting beef and dairy production [31]. Extensive areas of these pastures are sown on Andisols, which are predominantly characterized by a low pH, low P availability and toxic levels of Al [32]. Consequently, the yield, quality, and persistence of ryegrass plants can be severely affected by these constraints. Accordingly, the development of P-efficient and Al-resistant ryegrass has become an essential requirement for grassland systems developed on Andisols. In our previous studies on ryegrass, we found that the expression of the phosphate transporters *LpPHT1*;*1* and *LpPHT1*;*4* was significantly up-regulated by the addition of Al under P deficiency, with a further increase being observed in the Al-tolerant cultivar [33]. Although these findings could suggest that Al tolerance in ryegrass is closely related to the efficient transport of P, to our knowledge, little is known about the other Al tolerance responses underlying the joint effect between Al excess and low P in this plant species. Therefore, the aim of this study was to determine the roles of growth traits, P use efficiency, OA exudation, and the expression of Al tolerance genes in improving the tolerance of ryegrass to simultaneous low-P and Al stresses.

## 2. Results

### 2.1. Phosphorus and Aluminium Concentrations and Contents

During the time-course experiment, the P concentration decreased in shoots and roots at least 2.4-fold when the P dose was reduced to 0.01 mM, irrespective of the Al supply (Table 1). Under both sufficient and deficient P conditions, the shoot P concentration decreased by about 1.5-fold as a consequence of the addition of Al after 7 days. In contrast, Al application significantly increased the root P concentration at optimal P supply, whereas no changes in the root P concentration were observed when Al was added to plants cultivated at low P (Table 1). As expected, Al supply steadily increased the root Al concentration in plants grown with either 0.1 mM P or 0.01 mM P (Table 1). At 7 days, the highest amount of Al was detected in roots when Al was applied to plants grown under P limitation (Table 1). Nevertheless, significantly lower amounts of Al were translocated from the roots to shoots, independent of the P condition (Table 1). In addition, the shoot Al content decreased by about 30% in plants subjected to P deficiency compared to those grown under optimal P after 7 days of Al application (Table 1).

### 2.2. Plant Growth Parameters

**Figure 1:**
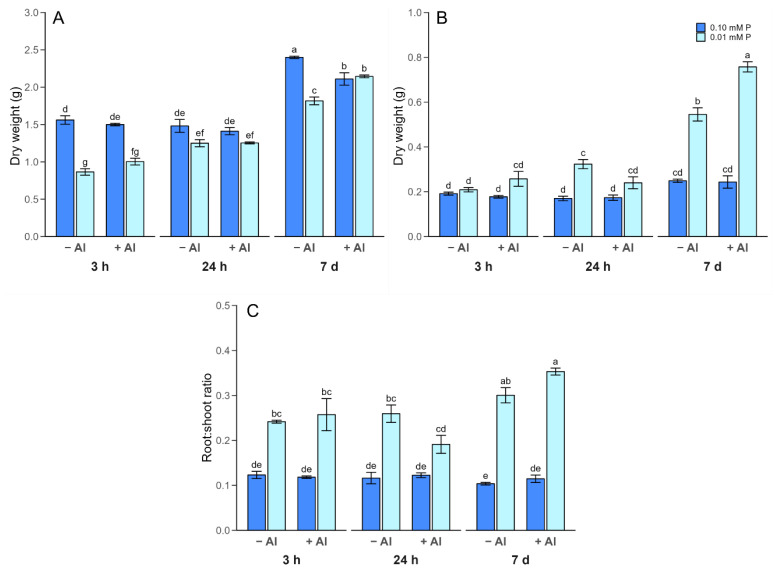
Time-dependent dry weight in shoots (**A**) and roots (**B**), and root/shoot ratio (**C**) in ryegrass plants hydroponically cultivated under different Al–P treatments. Values represent the mean (n = 3) ± SE. Different letters indicate statistically significant differences (Tukey’s HSD at *p* ≤ 0.05) among treatments over time-course experiment.

**Figure 2:**
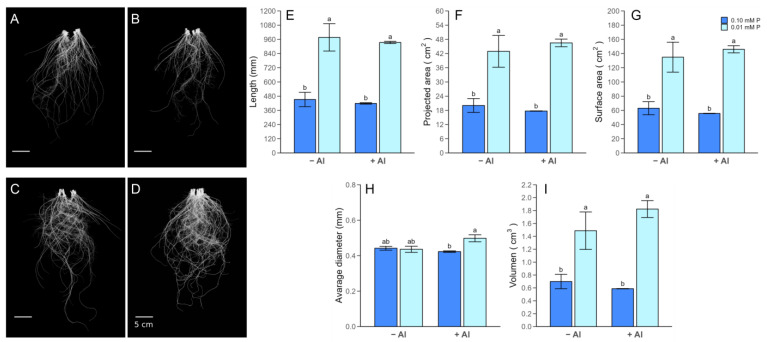
Root images and root morphology traits were assessed after 7 days of Al exposure under optimal P and P deficiency conditions. Images of ryegrass root system grown under (**A**) 0.1 mM of P and 0 mM of Al, (**B**) 0.1 mM of P and 0.2 mM of Al, (**C**) 0.01 mM of P and 0 mM of Al, and (**D**) 0.01 mM of P and 0.2 mM of Al. Changes in the root morphology were measured: (**E**) length (mm), (**F**) projected area (cm^2^), (**G**) surface area (cm^2^), (**H**) average diameter (mm), and (**I**) volume (cm^3^). Values represent the mean (n = 3) ± SE. Different letters indicate statistically significant differences (Tukey’s HSD at *p* ≤ 0.05) among P-Al treatments.

In general, P limitation reduced the production of shoot dry matter in plants without Al supply (Figure 1A). Even though the plant biomass was not affected by Al application in the short term (3 and 24 h), an increase in the shoot biomass was observed in plants subjected to 0.01 mM of P in combination with 0.2 mM of Al by 7 days (Figure 1A,B). The root dry weight was also augmented by low P addition, with a further increase being observed after 7 days of Al exposure (Figure 1B). In addition, independent of the Al added, P-deficient plants displayed higher root/shoot ratios than those grown under sufficient P (Figure 1C). Changes in the components of the root system architecture (RSA) were also induced by the P and Al treatments at 7 days (Figure 2). Compared with the plants supplied with an adequate P dose, the root length (RL), root volume (RV), projected root area (PRA) and root surface area (RRSA) increased by about 2-fold under low-P conditions both with and without Al supply. Despite the average root diameter (ARD) not showing differences among P treatments, the application of 0.2 mM of Al increased this RSA component by about 15% in P-deficient roots (Figure 2H).

### 2.3. Phosphorus Use Efficiency

**Figure 3:**
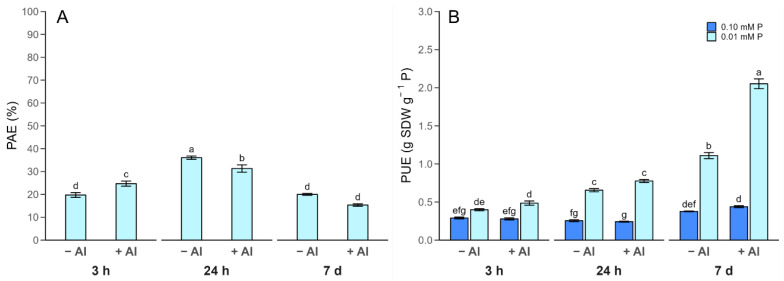
Time-dependent phosphorus acquisition efficiency (PAE; (**A**)) and phosphorus utilization efficiency (PUE; (**B**)) in ryegrass plants hydroponically cultivated under different Al–P treatments. Different letters indicate statistically significant differences (Tukey’s HSD at *p* ≤ 0.05) among treatments over time-course experiment.

To delve deeper into the influence of Al stress on the plant P status mainly under P-limited conditions, the P use efficiency expressed as the P acquisition efficiency (PAE) and the internal utilization efficiency (PUE) were calculated (Figure 3). Accordingly, the relative difference in shoot P acquired under P-deficient conditions versus a sufficient P state, known as PAE, decreased in response to Al addition after 7 days (Figure 3A). In contrast, the PUE, estimated as the shoot biomass produced per unit of P accumulated in the shoots, increased by about 1.8-fold when Al was applied to P-deficient plants for 7 days (Figure 3B). Under an adequate P regime, no changes in the PUE were triggered by Al during the time course.

### 2.4. Organic Acid in Root Exudates

**Figure 4:**
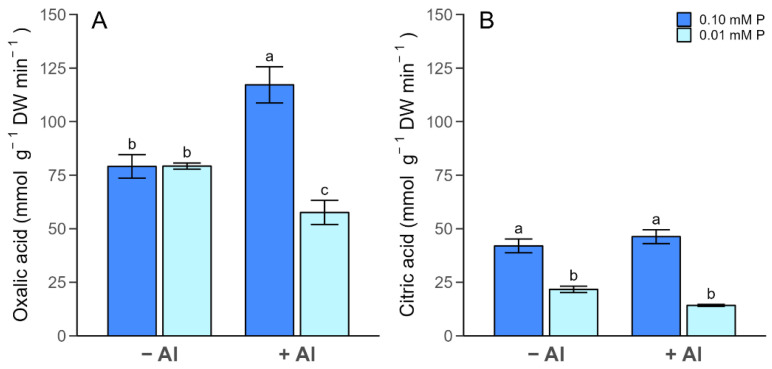
Oxalic acid concentration (**A**) and citric acid concentration (**B**) in root exudates of ryegrass plants hydroponically cultivated under different Al and P treatments. Different letters indicate statistically significant differences (Tukey’s HSD at *p* ≤ 0.05) among treatments.

Under different P treatments, the carboxylate exudation was also determined after 7 days of Al application (Figure 4). In general, major exudation rates of oxalate compared with citrate were detected in ryegrass plants (see Supplementary Materials). Likewise, the highest oxalate and citrate exudation rates were found under optimal P conditions with both Al supplies (Figure 4A,B). A significant decrease in oxalate exudation was triggered by Al under P deficiency, whereas it increased in plants subjected to Al in combination with sufficient P (Figure 4A). In contrast, Al did not induce changes in citrate exudation irrespective of the P addition level (Figure 4B).

### 2.5. Gene Expression Analyses

**Figure 5:**
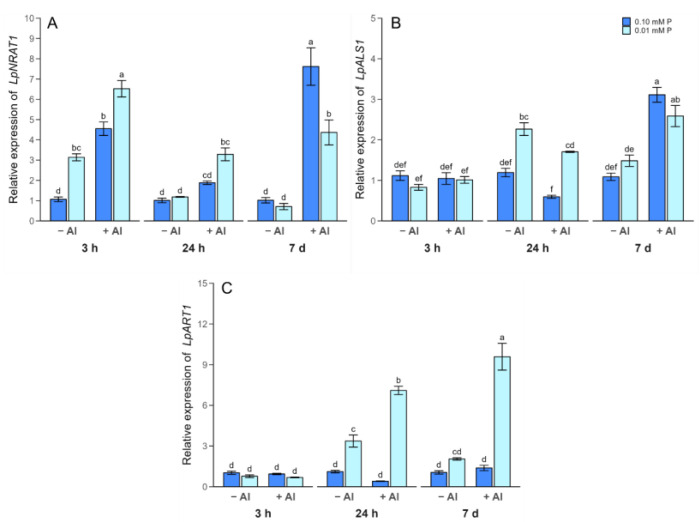
Time-dependent relative expression of *LpNRAT1* (**A**), *LpALS1* (**B**) and *LpART1* (**C**) in ryegrass plants hydroponically cultivated under different Al and P treatments. Different letters indicate statistically significant differences (Tukey’s HSD at *p* ≤ 0.05) among treatments over time-course experiment.

The expression pattern of the key Al-responsive genes involved in internal Al detoxification (*LpNRAT1*, *LpALS1* and *LpART1*) were also analyzed in the roots of ryegrass plants exposed to Al stress under low and optimal P conditions. The expression pattern of *LpNRAT1* was highly induced by Al exposure under P deficiency regardless of the time (Figure 5A). Nevertheless, the early (3 h) up-regulation of *LpNRAT1* was also observed without the application of Al to P-deficient plants. In addition, at 7 days, a significant increase in *LpNRAT1* expression was detected in the roots of plants receiving Al in combination with an optimal P dose (Figure 5A). Although Al supply did not induce changes in the expression of *LpALS1* in the short term, the transcript levels of this gene were greater under P limitation than at optimal P after 24 h (Figure 5B). In contrast, in the long term, Al addition significantly increased the expression pattern of *LpALS1* by about 1.8-fold and 2.8-fold at P deficiency and P sufficiency, respectively (Figure 5B). On the other hand, Al application for 24 h and 7 d increased the expression of *LpART1* only in plants subjected to low P. The up-regulation of this gene was also observed when plants were grown in the absence of Al and under limited P during 24 h (Figure 5C).

### 2.6. Principal Component Analysis (PCA)

**Figure 6:**
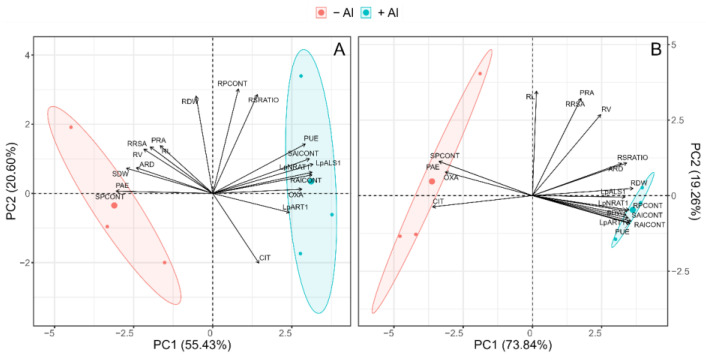
Principal component analysis (PCA) of different traits in ryegrass plants hydroponically cultivated and grown under optimal (0.1 mM of P; (**A**)) and deficient (0.01 mM of P; (**B**)) P conditions. Abbreviations: shoot P content (SPCONT), root P content (RPCONT), shoot dry weight (SDW), root dry weight (RDW), root/shoot ratio (RSRATIO), root length (RL), root volume (RV), projected root area (PRA), root surface area (RRSA), average root diameter (ARD), phosphorus acquisition efficiency (PAE), phosphorus utilization efficiency (PUE), oxalate exudation (OXA), citrate exudation (CIT), *LpNRAT1* expression (LpNRAT1), *LpALS1* expression (LpALS1), and *LpART1* expression (LpART1). The confidence interval is depicted by different Al treatments of ryegrass plants; 0 mM of Al (−Al) and 0.2 mM of Al (+Al).

PCA was performed using all the parameters measured after 7 days of Al exposure and separated per each P level (Figure 6). At both P conditions, the confidence ellipsoids showed a clear separation of two well-defined groups for Al treatments. Under the optimal P condition, the first two principal components explained 76.03% of the total variance, with 55.43% and 20.60% in the first (PC1) and second (PC2) principal components, respectively (Figure 6A). In PC1, the variables making the most positive contributions were LpALS1, LpNRAT1, RAlCONT and SAlCONT. Such variables were associated with the 0.2 mM of Al treatment. The main variables that contributed negatively to PC1 under an adequate P dose were SPCONT and PAE, which were associated with the absence of Al supply.

Conversely, under limited P conditions, the first two principal components explained 93.10% of the total variance, with 73.84% and 19.26% in the first (PC1) and second (PC2) principal components, respectively (Figure 6B). Accordingly, the variables showing a major contribution to the PCA1 under Al toxicity were RDW, RPCONT, SAlCONT, RAlCONT, PUE, and Al-responsive genes (Figure 6B). In contrast, SPCONT, PAE, OXA, and CIT were linked to non-Al application.

## 3. Discussion

It is well known that Al toxicity and P deficiency are the most limiting factors for agricultural production in acidic soils. However, the influence of Al stress along with P limitation on plant performance is still not fully understood. Here, we focused on elucidating the role of growth traits, P use efficiency, carboxylate exudation, and the expression of Al-responsive genes in improving tolerance to concurrent Al and low-P stress in ryegrass.

In general, shoot and root P accumulation was not affected by short-term (3 h and 24 h) Al application, irrespective of the P level. Nevertheless, an increase in the root P content was observed in P-deficient plants after 7 days of Al treatment (Table 1). The improvement in root P accumulation was also accompanied by an increase in the root Al content, while less Al was accumulated in shoots under the low-P and Al toxicity treatment. Although changes in the root system in this study, including the increase in root biomass, were mostly attributed to P deficiency, Al stress also augmented the root biomass and the average root diameter (Figure 1C and Figure 2H). Thus, the improvement in root traits triggered by Al under P limitation could be related to the higher accumulation of P and Al in the roots. Accordingly, our results suggest an immobilization of Al by P in root tissues, which has been proposed as a crucial mechanism of Al detoxification in plants [3,34]. Moreover, consistent with our findings, large amounts of Al in roots and lower translocation to the shoots of an Al-tolerant ryegrass cultivar were previously reported by Parra-Almuna et al. (2018) [33]. We also observed an increase in shoot DW in P-deficient plants after 7 days of Al exposure. In this line, as reviewed by Ofoe et al. (2023) [13], Al-induced growth stimulation has been reported in some plant species adapted to acidic soils. Although the specific mechanism by which Al could enhance plant growth remains elusive, the increase in shoot biomass induced by Al in this study could be related to a hormetic response used by plants to cope with Al-induced stress, as described for selenium toxicity [35].

The increase in tolerance needed by plant genotypes to overcome Al stress under P deficiency has been attributed to the highly efficient acquisition and utilization of P [28,29]. In this sense, a pleiotropy between Al resistance and enhanced P uptake via improved root morphological traits has been suggested by Magalhaes et al. (2018) [26]. We recently found that ryegrass cv. “24Seven” displayed an efficient remobilization of P within shoots that was mediated by P transporters [36]. This leads to an improved capacity for using acquired P for biomass production (PUE), mainly under low-P conditions. Interestingly, in our study, the highest PUE was found in P-deficient plants under long-term Al stress (Figure 3B). Furthermore, as supported by PCA, a strong association between Al toxicity and improved PUE was observed when P was limited (Figure 6B), suggesting that this cultivar has developed a more efficient mechanism to utilize P and simultaneously survive under Al stress.

The most common plant mechanism used to cope with either Al toxicity or P limitation is the secretion of OAs [3,8]. The OAs exuded by plant roots form non-toxic compounds with Al and release P in the rhizosphere, leading to improved P availability and the alleviation of Al toxicity [16]. Oxalate and citrate were the predominant carboxylates found in the ryegrass exudates (Figure S1), which was consistent with previous findings regarding the conditions of manganese toxicity in ryegrass plants [37,38]. The highest level of oxalate concentration was found in the optimal P-treated plants exposed to Al stress, confirming that oxalate exudation occurs under Al toxicity (Figure 4). Nevertheless, a decrease in the oxalate and citrate concentration was observed when plants were grown under both P limitation and Al toxicity (Figure 4). These results may indicate that the release of OAs outside the root tissue is not the main strategy used by ryegrass plants to counteract the joint impact of Al and low-P stress. In fact, OAs can act in the internal sequestration of Al^3+^ and release P from Al–P complexes within the cells [15,22]. Moreover, other compounds (e.g., phenolic compounds), rather than OAs, could alleviate Al toxicity and low P in plants [3]. Further research is necessary to confirm the role of OAs in ryegrass plants subjected to simultaneous low-P and Al excess.

The molecular traits associated with the key Al-responsive genes were also analyzed to further elucidate other mechanisms common to both stresses in ryegrass. In this context, improved Al tolerance in rice has been linked to the function of the key transcription factor OsART1 (aluminum resistance transcription factor 1) [39,40,41]. OsART1 modulates the expression of at least 31 genes implicated in the external and internal detoxification of Al [39]. We found that the expression of *LpART1* was highly upregulated in response to P limitation, especially when Al was added (Figure 5C), suggesting that low-P stress signals may induce the regulation of *LpART1* at the transcriptional level. Similarly, Mora-Macías et al. (2018) [42] and Godon et al. (2019) [18] found that P deficiency increased the accumulation of AtSTOP1 (sensitive to proton rhizotoxicity 1), which is the homolog of OsART1 in Arabidopsis. AtSTOP1 induces the expression of malate and citrate transporter genes, which in turn improve P acquisition. These studies have revealed the pleiotropic role of AtSTOP1 in conferring Al and low-P stress tolerance in Arabidopsis. However, the specific mechanism underlying the regulation of *LpART1* is still unclear, and deserves to be investigated.

Among the ART1-regulated genes, the Al transporter genes *NRAT1* and *ALS1* play an important role in the internal Al detoxification mechanism [23,25]. NRAT1 works in tandem with ALS1 to remove Al from the cell wall and sequester it into the vacuole, conferring Al tolerance to rice [43]. In our study, the time-course experiment showed that the expression of *LpNRAT1* was rapidly induced (3 h) in response to Al stress, whereas *LpALS1* expression exhibited upregulation after 24 h of Al exposure (Figure 5A,B), thus providing the first evidence that these transporters are also involved in Al uptake and Al sequestration in ryegrass. Consistent with our results, higher *NRAT1* and *ALS1* expression has been shown to be responsible for Al tolerance in rice [41,43,44] and *Hydrangea macrophylla* [22], suggesting that both Al transporters may be involved in internal Al detoxification in ryegrass. Interestingly, the transcript levels of both *LpNRAT1* and *LpALS1* were enhanced by P limitation (Figure 5A,B). Although the higher Al content in P-deficient ryegrass roots could be responsible for the upregulation of *LpNRAT1* and *LpALS1*, we also observed an increase in the expression of these genes in plants grown at low P without Al stress. In addition, we have previously observed an increase in the expression of the phosphate transporters (i.e., *LpPHT1*;*1* and *LpPHT1*;*4*) induced by Al in an Al-tolerant ryegrass cultivar, leading to the maintenance of optimal P levels under Al stress [33]. This behavior indicates that pleiotropy is a genetic link between Al resistance and P efficiency [26]. Indeed, the PCA also showed a positive relationship between the expression of all Al-responsive genes and the efficiency of P utilization (Figure 6A,B), suggesting that this ryegrass cultivar displayed an improved PUE and higher internal Al tolerance.

Taken together, our findings provide new insights into the role of growth traits, P use efficiency, carboxylate exudation, and the expression of Al-responsive genes in coping with combined Al and low-P stress in ryegrass. Indeed, the increased P-mediated Al immobilization in the roots, efficient reduction in Al translocation to the shoots, improved PUE, and coordinated function of genes involved in Al uptake and sequestration were found to be the critical strategies used by ryegrass plants to counteract the joint impact of Al toxicity and P limitation. In addition, this study has provided the first evidence of Al-responsive genes in ryegrass and their contribution to Al detoxification under combined Al and low-P stress. Nevertheless, future studies using comparative RNA-seq analysis are needed to better understand the genetic responses leading to improved tolerance mechanisms for both P deficiency and Al toxicity in ryegrass.

## 4. Materials and Methods

### 4.1. Plant Material and Growth Conditions

Seeds from ryegrass, *Lolium perenne* L. cv. 24Seven (New Zealand origin and provided by SG-2000 Company, Cajón, Chile), were rinsed with 2% *v*/*v* sodium hypochlorite for 10 min, washed several times with distilled water, and then germinated on moist filter paper in a growth chamber at 21 °C. After 10 days of germination, the seedlings were transferred to continuously aerated hydroponic culture pots (7 L) using a basal nutrient solution proposed by Taylor and Foy (1985) [45], which contained the following: 1270 µM of Ca(NO_3_)_2_, 650 µM of KNO_3_, 120 µM of MgSO_4_, 100 µM of K_2_HPO_4_, 2.4 μM of MnSO_4_, 1000 µM of NH_4_NO_3_, 6.6 μM of Na_2_B_4_O_7_, 150 μM of Mg(NO_3_)^2^, 0.1 μM of (NH_4_)_6_Mo_7_O_24_, 0.6 μM of ZnSO_4_, 0.2 μM of CuSO_4_, 17.9 μM of Fe-EDTA and 46 μM of NaCl. Plants were grown in a greenhouse under controlled conditions (16 h/8 h (light/darkness) photoperiod at 20 °C and 75–80% relative humidity). The seedlings were adapted for one week in the nutrient solution adjusted to pH 4.8. After the acclimation period, the plants were subjected to two P treatments (0.1 mM, P-optimal [+P]; and 0.01 mM of P, P-deficiency [−P]; supplied as K_2_HPO_4_) for 21 days. Potassium (K) was supplied as KCl to maintain equal concentrations of K in the solution. After 21 days, two Al doses were applied (0 mM [−Al]; and 0.2 mM [+Al]; supplied as AlCl_3_) in combination with P doses, in a completely randomized factorial design with three replicates per treatment, as previously reported by Parra-Almuna et al. (2018) [33]. During the experiment, the pH of the nutrient solution was checked daily and adjusted to 4.8 using diluted HCl or NaOH; the nutrient solution was replaced every 4 days. Shoot and root tissues were harvested after short-term (3 h and 24 h) and long-term (7 days) Al treatment. The collected plant material was quickly stored until further biochemical and molecular analyses.

### 4.2. Phosphorus and Al Determinations

For the determination of the P and Al concentrations, shoot and root samples (0.25 g) were ashed in a muffle at 500 °C for 10 h, then digested with 2 M HCl and filtered with quantitative filter paper. The P concentration was measured using the molybdovanadate method at 466 nm and the Al concentration was determined using flame atomic absorption spectrophotometry (FAAS) at 324.7 nm, both according to the methodology described by Sadzawka et al. (2004) [46]. Two reference samples with known P and Al concentrations were included for each analytical run as internal controls. The P and Al contents were determined by multiplying the P concentration in plant tissues by the biomass produced (DW).

The phosphorus acquisition efficiency (PAE) and phosphorus utilization efficiency (PUE) were calculated as previously described by Pontigo et al. (2023) [36]. Briefly, the PAE was calculated as the ratio between the shoot P content under P-deficient conditions and the shoot P content under optimal P supply; meanwhile, the PUE was determined as the ratio between the shoot DW and shoot P concentration.

### 4.3. Plant Growth Traits

At each harvest time, subsamples of fresh plant material were oven-dried at 65 °C for 48 h to obtain the dry weight (DW). The root/shoot DW ratio was also calculated. In addition, the root subsamples were separated from the shoots after 7 days of Al application. The separated roots were washed 3–4 times with deionized water and then the whole root system was scanned with a root system scanner (1680; Epson, Long Beach, CA, USA) at 300 dpi. Root architecture (RSA) traits such as the total root length (cm), root surface area (cm^2^), root volume (cm^3^), average root diameter (mm), and projected root area (cm^2^) were measured from four plants per biological replicate using the WinRHIZO™ image analysis system (Regent Instruments Inc., Quebec, ON, Canada).

### 4.4. Determination of Root Exudates

Root exudates were collected after 7 days of Al treatment and analyzed as described by Rosas et al. (2007) [37]. Briefly, the intact ryegrass plants grown in the nutrient solution were removed and the roots were rinsed with deionized water. The whole root system was placed in 50 mL of ultrapure water with aeration for 2 h to avoid the microbial degradation of organic acid anions [35]. Next, the root exudates were concentrated via lyophilization. The lyophilized products were resuspended in 1000 µL of ultrapure water, and then filtered through a 0.22 µm filter. Samples were analyzed by high-performance liquid chromatography with a diode array detector (HPLC-DAD) equipped with an LC-20AT quaternary pump, a DGU-20A5R degassing unit, a CTO-20A oven, a SIL-20a autosampler, and an array of a UV–visible detector diode (SPD M20A) (Shimadzu, Tokyo, Japan). Chromatographic analysis was performed using a Zorbax Eclipse Agilent C18 column (250 mm × 4.6 mm, 5 μm) and a Novapak Waters C18 precolumn (22 mm × 3.9 mm, 4 μm) at 30 °C based on the method reported by Parada et al. (2019) [47] with modifications. The samples were eluted with a mobile phase of 200 mM of ortho-phosphoric acid solution at pH 2.1, at a flow rate of 1 mL min^−1^. Quantification was performed at 210 nm by external calibration.

### 4.5. Expression Analyses of Al-Responsive Genes

The expression patterns of the internal Al tolerance genes were evaluated in the roots of the ryegrass plants grown as above. Total RNA was extracted using the NucleoSpin^®^ RNA Plant Kit, according to the manufacturer’s instructions (Macherey-Nagel, Düren, Germany). The concentration and purity of the RNA were checked spectrophotometrically using a NanoDrop™ (Thermo Scientific, Wilmington, DE, USA). The RNA concentration was adjusted to 1 µg for the synthesis of the first strand of cDNA using a High-Capacity cDNA Reverse Transcription Kit (Invitrogen, Carlsbad, CA, USA). The gene expression level was analyzed by quantitative real-time reverse transcription polymerase chain reaction (qRT-PCR) on a qPCR Step One™ Plus (Applied Biosystems, Foster City, CA, USA) and with the PowerUp™ SYBR™ Green Master Mix Kit (Agilent, Santa Clara, CA, USA). Specific primers were designed using Primer3 (V. 0.4.0) software (Table 2) to amplify the following genes: *LpNRAT1* (GeneBank accession XM_051339444), *LpALS1* (GeneBank accession OR770646) and *LpART1* (GeneBank accession OR770647). Two housekeeping genes, eukaryotic elongation factor 1 alpha *eEF1α(h)* (GeneBank accession GO924753) and *eEF1α(s)* (GeneBank accession GO924801), were used as internal controls (Table 2). All qRT-PCRs were performed using three biological replicates with three technical replicates. The relative expression levels of the Al resistance genes were analyzed with the comparative threshold cycle method 2^−∆∆CT^ [48].

### 4.6. Statistical Analyses

The statistical analyses and figures were performed and created using R software (version 4.2.1). A two-way analysis of variance (ANOVA) was conducted to test for significant differences between the measurements of each experimental variable. For variables showing significant differences, means were compared using the Tukey HSD multiple range test with the “agricolae” package v. 1.3.5. Additionally, the dataset for the variables at 7 days was segregated by P levels, and both datasets underwent principal component analysis (PCA). Confidence ellipses (group means) based on differences in the Al treatments were also generated using the “FactoMiner” package v. 2.7 and “factorextra” v.1.0.7.

## Figures and Tables

**Table 1:** Aluminum and P concentration and content in the shoots and roots of ryegrass plants hydroponically cultivated under different Al–P treatments. Values represent the mean (n = 3) ± SE. Different letters indicate statistically significant differences (Tukey’s HSD at *p* ≤ 0.05) among treatments over time-course experiment.

**Treatment (mM)**	**Time**	**P Concentration** **(g kg^−1^)**		**P Content** **(mg pot^−1^)**	
		**Shoot**	**Root**	**Shoot**	**Root**
0 Al–0.01 P	3 h	2.17 ± 0.12 e	2.61 ± 0.01 d	1.66 ± 0.09 d	0.55 ± 0.03 f
	24 h	1.91 ± 0.03 efg	1.97 ± 0.08 e	2.69 ± 0.05 d	0.64 ± 0.03 f
	7 d	1.64 ± 0.01 fg	1.1 ± 0.11 f	2.77 ± 0.20 d	0.64 ± 0.02 f
0.2 Al–0.01 P	3 h	2.07 ± 0.06 ef	2.68 ± 0.11 d	2.08 ± 0.09 d	0.68 ± 0.07 ef
	24 h	1.62 ± 0.03 g	2.25 ± 0.09 de	2.33 ± 0.12 d	0.54 ± 0.05 f
	7 d	1.05 ± 0.04 h	1.29 ± 0.05 f	2.28 ± 0.08 d	0.98 ± 0.05 de
0 Al–0.1 P	3 h	5.38 ± 0.14 c	6.36 ± 0.07 bc	8.40 ± 0.38 c	1.22 ± 0.03 cd
	24 h	5.79 ± 0.06 bc	6.24 ± 0.06 c	7.46 ± 0.19 c	1.06 ± 0.06 cd
	7 d	6.37 ± 0.04 a	6.15 ± 0.02 c	14.86 ± 0.44 a	1.53 ± 0.05 ab
0.2 Al–0.1 P	3 h	5.41 ± 0.21 bc	7.69 ± 0.04 a	8.11 ± 0.23 c	1.37 ± 0.05 abc
	24 h	5.83 ± 0.05 b	7.58 ± 0.06 a	7.68 ± 0.65 c	1.31 ± 0.09 bc
	7 d	4.81 ± 0.05 d	6.74 ± 0.24 b	10.16 ± 0.50 b	1.63 ± 0.12 a
**Treatment (mM)**	**Time**	**Al concentration** **(g kg^−1^)**		**Al content** **(mg pot^−1^)**	
		**Shoot**	**Root**	**Shoot**	**Root**
0 Al–0.01 P	3 h	0.07 ± 0.00 de	0.09 ± 0.00 e	0.06 ± 0.00 ef	0.02 ± 0.00 e
	24 h	0.08 ± 0.00 cd	0.09 ± 0.00 e	0.09 ± 0.01 de	0.03 ± 0.00 e
	7 d	0.09 ± 0.00 c	0.13 ± 0.00 e	0.10 ± 0.00 cd	0.08 ± 0.01 de
0.2 Al–0.01 P	3 h	0.07 ± 0.00 de	1.72 ± 0.06 d	0.07 ± 0.00 def	0.44 ± 0.06 bc
	24 h	0.09 ± 0.00 c	3.11 ± 0.30 c	0.14 ± 0.01 c	0.75 ± 0.12 bc
	7 d	0.12 ± 0.00 b	5.62 ± 0.35 a	0.26 ± 0.00 b	4.12 ± 0.18 a
0 Al–0.1 P	3 h	0.02 ± 0.00 f	0.06 ± 0.00 e	0.04 ± 0.00 f	0.01 ± 0.00 e
	24 h	0.06 ± 0.00 e	0.12 ± 0.00 e	0.08 ± 0.00 def	0.01 ± 0.00 e
	7 d	0.11 ± 0.00 b	0.12 ± 0.00 e	0.11 ± 0.00 cd	0.03 ± 0.00 e
0.2 Al–0.1 P	3 h	0.03 ± 0.00 f	1.75 ± 0.12 d	0.04 ± 0.00 f	0.44 ± 0.05 bc
	24 h	0.09 ± 0.00 c	4.41 ± 0.09 b	0.10 ± 0.01 cd	0.41 ± 0.02 cd
	7 d	0.18 ± 0.00 a	3.22 ± 0.36 c	0.38 ± 0.02 a	0.77 ± 0.01 b

**Table 2:** Primers used for analysis of Al resistance genes from ryegrass plants.

Gene Name *	Forward Primer (5′–3′)	Reverse Primer (5′–3′)
*LpNRAT1*	ATGTTCACCATGGCAGGATGCT	ACTAGGGCAGAGTGCAAGAACAAG
*LpALS1*	ACGCAGTGCTTCTGAAAGGT	CAGTTTGCACAGCTCTTCGG
*LpART1*	ACCCCTCGGACTGATCTTCT	AGATAAGGTGGCTCACGCAG
*eEF1α(h)*	ATG TCT GTT GAG CAG CCT TC	GCG GAG TAT ATA AAG GGG TAGC
*eEF1α(s)*	CCG TTT TGT CGA GTT TGG T	AGC AAC TGT AAC CGA ACA TAGC

* *LpNRAT1*, Al transporter 1; *LpALS1*, (ABC) transporter Al-sensitive 1; *LpART1*, Al resistance transcription factor 1; *eEF1α*, eukaryotic elongation factor 1 alpha h-s.

## Data Availability

Data are contained within the article and Supplementary Materials.

## Supplementary Materials

The following supporting information can be downloaded at https://www.mdpi.com/article/10.3390/plants13070929/s1, Figure S1: HPLC-DAD chromatogram (210 nm) of organic acids in ryegrass plants growing under different P-Al treatments.
