# Supplementary material for: Dissecting the Roles of Phosphorus Use Efficiency, Organic Acid Anions, and Aluminum-Responsive Genes under Aluminum Toxicity and Phosphorus Deficiency in Ryegrass Plants
Source: Plants (Basel). 2024 Mar 23;13(7):929. doi: 10.3390/plants13070929 (PMC11013041; doi:10.3390/plants13070929)
Supplement: Supplementary file 1 [file plants-13-00929-s001.zip › plants-2872710-supplementary.pdf]

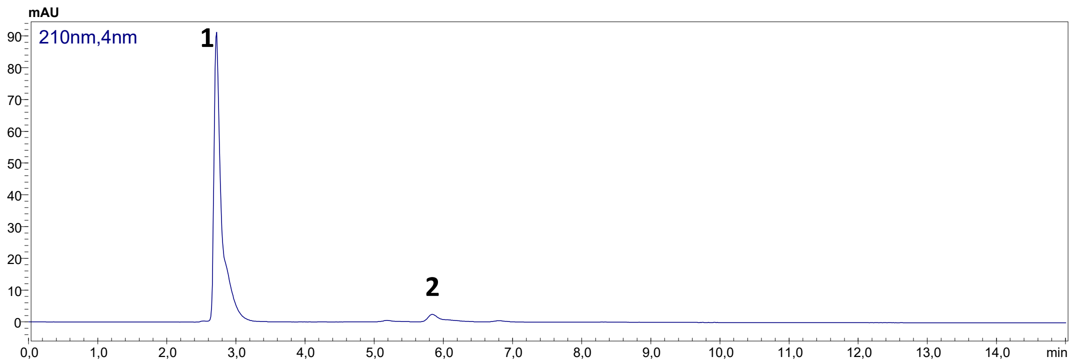

Figure S1. HPLC-DAD chromatogram (210 nm) of organic acids, (1); Oxalic acid and (2); Citric acid in ryegrass plants growing under different P-Al treatments
